# Investigation of post-therapeutic image-based thyroid dosimetry using quantitative SPECT/CT, iodine biokinetics, and the MIRD’s voxel S values in Graves’ disease

**DOI:** 10.1186/s40658-020-0274-7

**Published:** 2020-01-28

**Authors:** Naotoshi Fujita, Yumiko Koshiba, Shinji Abe, Katsuhiko Kato

**Affiliations:** 1 Department of Radiological Technology, Nagoya University Hospital, 65 Tsurumai-cho, Showa-ku, Nagoya, 466-8560 Japan; 2 Department of Radiological and Medical Laboratory Sciences, Nagoya University Graduate School of Medicine, 1-1-20 Daiko-Minami, Higashi-ku, Nagoya, 461-8673 Japan

**Keywords:** Absorbed dose, Graves’ disease, Internal radionuclide dosimetry, Radioiodine therapy, SPECT/CT, Thyroid

## Abstract

**Background:**

Before radioiodine therapy for Graves’ disease, the estimated thyroid-absorbed dose is calculated based on various clinical parameters. However, the actual accumulation of iodine in the thyroid during radioiodine therapy is not determined. We validated the feasibility of post-therapeutic image-based thyroid dosimetry through quantitative single-photon emission computed tomography (SPECT) imaging and thyroid biokinetics and expanding the Medical Internal Radiation Dose Committee’s (MIRD) voxel dosimetry guidelines.

**Methods:**

Forty-three patients with Graves’ disease who underwent radioiodine therapy were chosen as subjects for this retrospective analysis. We acquired patients’ SPECT images 24 h after oral administration. SPECT images were quantified using system volume sensitivity to calculate time-integrated activity coefficients on a voxel basis. Absorbed dose was obtained by convolving MIRD guideline voxel S values with time-integrated activity coefficients. To determine accuracy, we compared the results obtained using the post-therapeutic image-based absorbed-dose method (*D̅*_image,PVC_) with absorbed doses calculated using the method described by the European Association of Nuclear Medicine (pre-therapeutic method; *D*_EANM_).

**Results:**

Using image-based dosimetry as post-therapeutic dosimetry, we visualized the local accumulation and absorbed dose distribution of iodine in the thyroid. Furthermore, we determined a strong correlation (Pearson’s correlation coefficient = 0.89) between both dosimetries, using the regression equation: *D̅*_image,PVC_ = 0.94 × *D*_EANM_ + 1.35.

**Conclusion:**

Post-therapeutic image-based doses absorbed in the thyroid resembled those of pre-therapeutic EANM method-based absorbed doses. Additionally, the post-therapeutic image-based method had the advantage of visualizing thyroid iodine distribution, thus determining local dose distributions at the time of treatment. From these points, we propose that post-therapeutic image-based dosimetry could provide an alternative to standard pre-therapeutic dosimetry to evaluate dose response.

## Background

Graves’ disease is an autoimmune disease of the thyroid, and the main treatment options are medication (beta-blockers or antithyroid drugs), radioiodine therapy (iodine-131), and thyroidectomy [1]. Radioiodine therapy for Graves’ disease has been a popular and effective treatment for more than 50 years [2, 3]. The therapeutic effect of radioiodine therapy for Graves’ disease relates to the amount of administered radioactivity, or *thyroid-absorbed dose* [4, 5]. Several methods for calculating absorbed dose [6–8] include Marinelli et al.’s [8] or the European Association of Nuclear Medicine (EANM) guideline’s methods [9, 10]. Using the EANM guidelines, absorbed dose is determined based on thyroid volume, radioiodine uptake (RIU), and effective iodine half-life. However, the actual accumulation of iodine in the thyroid at the time of radioiodine therapy can only be estimated because these calculations use formulae to determine administered radioactivity before treatment. Furthermore, these formulae assume homogeneous uptake of iodine in the thyroids of patients with Graves’ disease.

An alternative to pre-therapeutic calculation, quantitative measurements using single-photon emission computed tomography (SPECT) visualize the actual uptake and distribution of iodine in the thyroid and calculate the absorbed dose of iodine-131 at the time of radioiodine therapy. If this image-based absorbed dose is calculated as post-therapeutic dosimetry, we can directly grasp the local accumulation and dose distribution of iodine-131 during treatment. Although previous studies have utilized radioactive tracers to measure the absorbed radiation doses of organs or tumors from SPECT images [11, 12], to the best of our knowledge, this approach is yet to be used to measure absorbed doses in the thyroid or for treating Graves’ disease.

In this study, we validated the feasibility of the post-therapeutic image-based dosimetry for Graves’ disease, applying quantitative SPECT measurements and thyroid biokinetics, thus expanding the Medical Internal Radiation Dose Committee’s (MIRD) voxel dosimetry guidelines [13–16].

## Methods

### Patients

Forty-five consecutive patients with Graves’ disease who underwent radioiodine therapy as outpatients from December 2014 to April 2018 were included in this retrospective analysis. Two of the 45 patients were excluded from the analysis for the following reasons: one due to insufficient dietary iodine restriction, and another for inaccurate measurements during the thyroid iodine-131 uptake test. A final 43 patients (7 males, 36 females) were included in this study. The average patient age was 51 ± 16 years. Because 5 patients underwent radioiodine therapy twice during this period, our analysis eventually included 48 cases. From 2 weeks before treatment, all patients were subjected to dietary iodine restriction and discontinued antithyroid drugs. In addition, patients underwent a non-enhanced neck CT examination (Aquilion 64 or Aquilion PRIME SP; Canon Medical Systems, Otawara, Japan) for thyroid volumetry. Contour extractions of the thyroid from CT images (5 mm slice thickness) were performed manually by one radiologist (nuclear medicine physician). The sum of the thyroid volume obtained from each slice was denoted as the total thyroid volume.

Subsequently, using a thyroid uptake system (AZ-800; Anzai Medical, Tokyo, Japan), which has a mono-photomultiplier tube and 2 in. (diameter) × 2 in. (thickness) NaI crystal, a thyroid iodine-131 uptake test (radioactivity = 3.7 MBq) was performed for all patients to predict the thyroid-absorbed dose during radioiodine therapy. RIU measurements were carried out 3, 24, 96, and 168 h after oral administration, and the effective iodine half-life was calculated by approximating RIUs after 24 h as a mono-exponential function. Using Marinelli et al.’s formula, administered radioactivity during radioiodine therapy was derived using the total thyroid volume, RIU at 24 h after administration, and effective iodine half-life [8]. Our hospital planned a thyroid-absorbed radiation dose of approximately 200–250 Gy. The nuclear medicine physician also adjusted the absorbed dose according to the clinical symptoms of each patient. In Japan, the administered radioactivity for outpatients with Graves’ disease has an upper limit of 500 MBq due to legal regulations. Therefore, we did not administer more than 500 MBq for these patients.

### EANM-based thyroid dosimetry (pre-therapeutic dosimetry); *D*_EANM_

We retrospectively calculated EANM guideline-based absorbed dose for each of the 48 cases. The iodine-131 uptake fraction of the thyroid at time *t* [*RIU*(*t*)] was modeled as a bi-exponential function using the 2-compartment model based on the iodine-131 biokinetics (Eq. 1) [9–11]:

1 $$ RIU(t)=\frac{k_{\mathrm{t}}}{k_{\mathrm{B}}-{k}_{\mathrm{T}}}\left({e}^{-{k}_{\mathrm{T}}t}-{e}^{-{k}_{\mathrm{B}}t}\right) $$

where *k*_t_ denotes the biokinetic transfer rate from the blood pool to the thyroid, *k*_B_ denotes the transfer rate from the blood pool to renal clearance or radioactive decay, and *k*_T_ denotes the rate of transfer from the thyroid to hormone excretion or radioactive decay. First, we obtained *k*_t_, *k*_B_, and *k*_T_ fitting the four RIU time points using the Levenberg-Marquardt method (GNU Octave software version 4.4.1) to Eq. 1 [10, 17]. Then, the absorbed dose *D*_EANM_ (Gy) was calculated using Eqs. 2 and 3 from the EANM guidelines [9–11] and Hänscheid’s methods [17]:

2 $$ {D}_{\mathrm{EANM}}\left[\mathrm{Gy}\right]=A\kern0.28em \overline{E}\kern0.24em {\int}_0^{\infty } RIU(t) dt/V=A\kern0.28em \overline{E}\kern0.28em {k}_{\mathrm{t}}/\left({k}_{\mathrm{B}}\kern0.28em {k}_{\mathrm{T}}\kern0.28em V\right) $$

3 $$ \frac{1}{\overline{E}}\left[\frac{\mathrm{MBq}\kern0.28em \mathrm{d}}{\mathrm{Gy}\kern0.28em \mathrm{g}}\right]=\frac{7.2}{(V)^{0.25}+18} $$

where *A* is the administered radioactivity (MBq), $\overline{E}$ is the mean deposition energy per iodine-131 decay (Eq. 3), and *V* is thyroid volume in milliliters obtained by neck CT images. Here, the density of the thyroid was assumed to be 1 g/mL.

### Image-based thyroid dosimetry (post-therapeutic dosimetry); *D*_image_

For our study, the post-therapeutic absorbed dose was calculated for each voxel on the basis of the MIRD’s guidelines for voxel dosimetry using SPECT images. Patients’ SPECT images were acquired using a Symbia T6 (Siemens Healthcare, Erlangen, Germany) 24 h after oral administration to confirm iodine uptake in the thyroid. SPECT images were acquired using the following parameters: high energy (HE) collimation, a 128 × 128 matrix, 20 s/projection, and total 60 projections (360° acquisition orbit). SPECT images were then reconstructed using ordered subset expectation maximization with depth-dependent three-dimensional resolution recovery (OSEM-3D; 15 subsets, 30 iterations, no post-filter) with CT-based attenuation correction and energy window-based scatter correction (15% main window centered at 364 keV, 15% sub window to touch the lower and upper of the main window). To calculate image-based absorbed doses, it was necessary to estimate the radioactivity of each voxel from SPECT images. Prior to dosimetry, we used a cylindrical phantom (16 cm diameter, 15 cm depth, 3016 mL volume) filled with uniform iodine-131 (78 MBq), to obtain the system volume sensitivity (*S*_vol_), which is the conversion factor used for calculating radioactivity from SPECT counts, according to the method described by Zeintl et al. (Eq. 4) [18, 19]:

4 $$ {S}_{\mathrm{vol}}\left[\frac{\mathrm{MBq}}{\mathrm{cps}}\right]=\frac{A_{\mathrm{phantom}}\kern0.28em {T}_{\mathrm{phantom}}\kern0.28em {V}_{\mathrm{voxel}}}{C_{\mathrm{phantom}}{V}_{\mathrm{phantom}}} $$

where *A*_phantom_ is the decay corrected activity (MBq), *T*_phantom_ is the scan duration (s), *V*_voxel_ is the voxel volume (mL), *C*_phantom_ is the mean count in the SPECT image, and *V*_phantom_ is the cylindrical phantom volume (mL).

Next, SPECT counts were converted into time-integrated activity coefficients in order to apply voxel S values (mGy MBq^−1^ sec^−1^), as recommended by the MIRD [13–16]. Radioactivity for each voxel (MBq/voxel) was obtained by dividing the acquired SPECT counts (*C*_thyroid_) of each point (*x*, *y*, *z*) by the scan duration (*T*_thyroid_) and multiplying by *S*_vol_. SPECT images were obtained 24 h after oral administration; therefore, radioactivity per voxel at oral administration [*A*_0_(*x*, *y*, *z*)] was calculated by dividing the SPECT counts by pre-therapeutic RIU (24 h) (Eq. 5):

5 $$ {A}_0\left(x,y,z\right)\left[\mathrm{MBq}\right]=\frac{C_{\mathrm{thyroid}}\left(x,y,z\right){S}_{\mathrm{vol}}}{T_{\mathrm{thyroid}}\kern0.24em RIU\left(24\kern0.28em \mathrm{hr}\right)} $$

Since radioactivity at time *t* was calculated by multiplying the administered radioactivity by *RIU*(*t*), time-integrated activity coefficients [$\overset{\sim }{A}$ (*x*, *y*, *z*)] were derived by integrating over the whole range (Eq. 6). Furthermore, we obtained absorbed doses for each voxel [*D*_image_(*x*,*y*,*z*)] by convolving the S value kernel (*k*_s_) of 11 × 11 × 11 voxels (33 × 33 × 33 mm) (Eq. 7). According to Peters et al. [20] and Bockisch et al. [21], there is little difference between RIU at therapeutic and test doses. Therefore, we used the test dose RIU for our calculations:

6 $$ \overset{\sim }{A}\left(x,y,z\right)\left[\mathrm{MBq}\cdot \sec \right]={\int}_0^{\infty }{A}_0\left(x,y,z\right)\kern0.28em RIU(t) dt $$

7 $$ {D}_{\mathrm{image}}\left(x,y,z\right)\left[\mathrm{Gy}\right]=\overset{\sim }{A}\left(x,y,z\right)\otimes {k}_{\mathrm{s}} $$

The thyroid-absorbed dose was obtained by extracting the thyroid region from the whole absorbed-dose image. Box plots for thyroid-absorbed dose distributions were then generated for all patients. To check the accuracy of *D*_image_ values, we compared them to the *D*_EANM_ values. SPECT images often have partial volume effects resulting from the deterioration of spatial resolution; therefore, we assumed that the absorbed dose calculated using our image-based dosimetry would also be influenced by these effects. When determining mean thyroid-absorbed doses, we acquired a weighting factor based on the geometry transfer matrix (GTM) concept (Eq. 8) [22] for thyroid volume; to correct the partial volume effect (called partial volume correction; PVC), we divided mean *D*_image_ in thyroid region, ${\overline{D}}_{\mathrm{image}}$, by this weighting factor, *W*_PVC_ (Eq. 9):

8 $$ {W}_{\mathrm{PVC}}=\frac{1}{V/{V}_{\mathrm{voxel}}}{\int}_{\mathrm{VOI}} RSF(r) dr $$

9 $$ {\overline{D}}_{\mathrm{image},\mathrm{PVC}}\kern0.28em \left[\mathrm{Gy}\right]=\frac{{\overline{D}}_{\mathrm{image}}}{W_{\mathrm{PVC}}} $$

where *V*_voxel_ is the voxel volume (mL) and ${\overline{D}}_{\mathrm{image},\mathrm{PVC}}$ is the partial volume corrected mean of *D*_image_ in the thyroid region, ${\overline{D}}_{\mathrm{image}}$. Before the PVC, we visually confirmed no iodine-131 uptake outside the thyroid in the SPECT/CT images. The thyroid region in the SPECT image was determined using a threshold equal to the CT-based thyroid volume, *V*. The spatial resolution of the SPECT image in the *x*, *y*, *z* direction was measured using a line source (1 mm diameter) to obtain a three-dimensional (3D) point spread function [23]. Then, a binary image was created by setting the volume of interest (VOI) corresponding to the thyroid volume in the SPECT image, in which a value of 1 was given to the area within the thyroid VOI and 0 corresponded to the area outside the VOI. Convolution of the binary image with the 3D point spread function blurs the region of interest in the range of 0–1 to create a distribution (the *regional spread function*; RSF) [22]. By comparing the RSF and the original VOI, it was possible to determine how many SPECT counts remained in the VOI or spill-out. The PVC based in the GTM corrects for spill-out from the thyroid VOI.

### Statistical analysis

The statistical software R (software package version 3.5.1 for Windows) was used in this study. Using single regression analysis, we derived a regression equation for comparing EANM-based absorbed doses (*D*_EANM_) and mean image-based absorbed doses that incorporate PVC (${\overline{D}}_{\mathrm{image},\mathrm{PVC}}$) or no PVC (${\overline{D}}_{\mathrm{image}}$). In addition, the Bland-Altman analysis was performed for comparison between *D*_EANM_ and both ${\overline{D}}_{\mathrm{image},\mathrm{PVC}}$ and ${\overline{D}}_{\mathrm{image}}$.

We used the Bonferroni-corrected Wilcoxon-Mann-Whitney test to assess the relationship between therapeutic outcomes (hypothyroidism, euthyroidism, and hyperthyroidism) and the thyroid-absorbed dose calculated from each method. *p* values < 0.05 were considered statistically significant.

## Results

Table 1 summarizes patient details, including thyroid volume, RIU at 24 h, effective iodine half-life, administered radioactivity, *D*_EANM_, and mean *D*_image_ with/without PVC (${\overline{D}}_{\mathrm{image}}$, ${\overline{D}}_{\mathrm{image},\mathrm{PVC}}$). Mean thyroid volume and RIU at 24 h were 32.7 ± 15.1 mL (range = 12.0–77.4) and 0.66 ± 0.16 (range = 0.19–0.94), respectively. Mean effective iodine half-life and administered radioactivity were 6.14 ± 1.31 days (range = 2.98–9.16) and 397 ± 94 MBq (range = 222–481), respectively. Mean *D*_EANM_ calculated by EANM-based thyroid dosimetry was 230.2 ± 56.7 Gy (range = 104.3–362.3). Compared to mean *D*_EANM_, ${\overline{D}}_{\mathrm{image}}$ and ${\overline{D}}_{\mathrm{image},\mathrm{PVC}}$ were 132.6 ± 32.2 Gy (range = 53.1–200.7) and 217.9 ± 59.8 Gy (range = 95.7–348.6), respectively.

**Table 1:** Summary of study participant details

Cases (male/female)	48 (7/41)
Age [years]	51 ± 16 (21–81)
Thyroid volume [mL]	32.7 ± 15.1 (12.0–77.4)
Ioiodine-131 uptake at 24 h	0.66 ± 0.16 (0.19–0.94)
Effective iodine half-life [days]	6.14 ± 1.31 (2.98–9.16)
Administered radioactivity [MBq]	397 ± 94 (222–481)
*D*_EANM_ [Gy]	230.2 ± 56.7 (104.3–362.3)
Mean *D*_image_ without PVC (${\overline{D}}_{\mathrm{image}}$) [Gy]	132.6 ± 32.2 (53.1–200.7)
Mean *D*_image_ with PVC (${\overline{D}}_{\mathrm{image},\mathrm{PVC}}$) [Gy]	217.9 ± 59.8 (95.7–348.6)

*Data are expressed as mean ± 1 standard deviation (SD), if appropriate, as maximum and minimum values

**Fig. 1:**
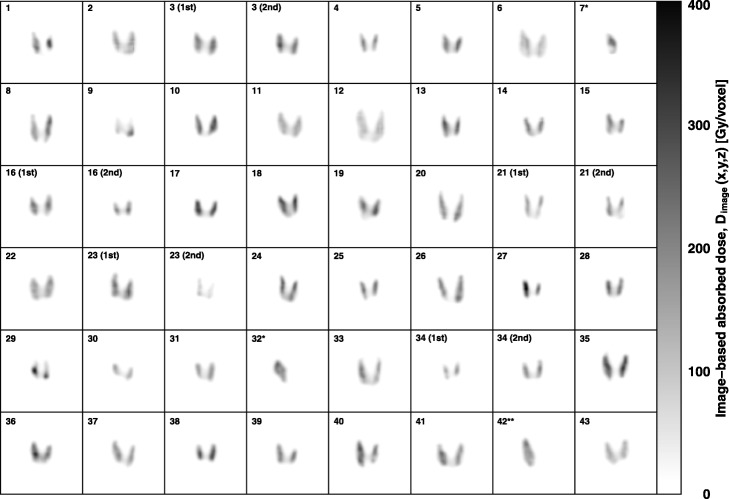
Absorbed dose image of thyroid obtained from each patient (coronal image). The accumulation of iodine in the thyroid was not completely homogeneous even for patients with Graves’ disease. Patient nos. 7 and 32 were imaged after undergoing subtotal thyroidectomy. Patient no. 42 had thyroid hemiagenesis

**Fig. 2:**
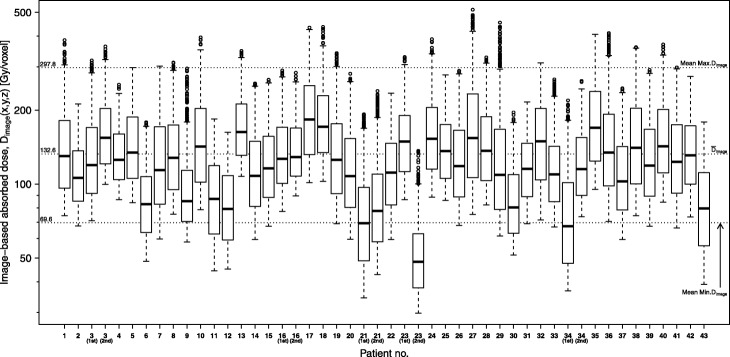
Image-based absorbed dose distribution of each patient (*D*_image_). Values were widely distributed, with the maximum absorbed dose approximately 2.5 times greater than the median

Visually, the absorbed dose distribution for thyroids (Fig. 1) appeared homogeneous in most cases, although some cases displayed a heterogeneous distribution (patient nos. 1, 2, 7, 9, and 29). Three cases (patient nos. 1, 2, and 7) became hypothyroidism, and the other two cases with more heterogeneous dose distribution (patient nos. 9 and 29) became euthyroidism. *D*_EANM_ is calculated as a single value due to the assumption that iodine-131 is homogeneously distributed in the thyroid. In comparison, *D*_image_ calculates the absorbed dose for each voxel, so the absorbed dose [*D*_image_(*x*,*y*,*z*)] varies from voxel to voxel. As a result, the thyroid-absorbed dose calculated using our image-based dosimetry was observed as a dose distribution (Fig. 2). Note that PVC is not applied to the dose distribution in Fig. 2. Mean maximum and minimum *D*_image_ values for the thyroid region were 297.8 ± 83.2 Gy (range = 136.6–512.9) and 69.6 ± 18.4 Gy (range = 29.8–107.5), respectively.

**Fig. 3:**
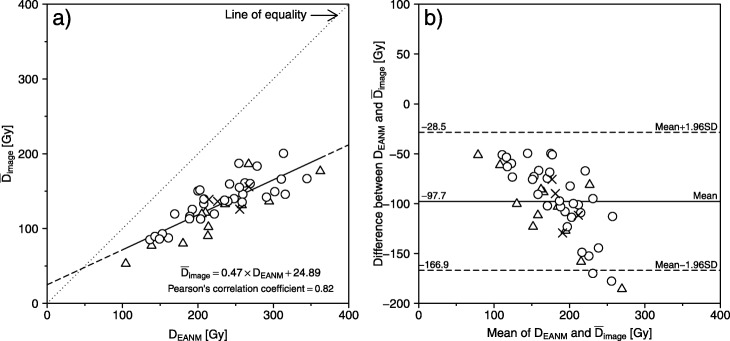
**a** Relationship between EANM-based absorbed dose (*D*_EANM_) and image-based absorbed dose (${\overline{D}}_{\mathrm{image}}$) without PVC, using the regression equation: ${\overline{D}}_{\mathrm{image}}$ = 0.47 × *D*_EANM_ + 24.89 (95% CIs of slope and *y*-intercept = 0.37–2.19 and 0.56–47.59, respectively). **b** The Bland-Altman analysis was performed between *D*_EANM_ and ${\overline{D}}_{\mathrm{image}}$. There were fixed bias (− 97.7 Gy [95% CI = − 107.9 to − 87.4]) and proportional bias (Pearson’s correlation coefficient = − 0.72 [95% CI = − 0.84 to − 0.55]). Note that each symbol in the figure represents three therapeutic outcomes: open circle, hypothyroidism; open triangle, euthyroidism; and cross, hyperthyroidism

Figure 3a shows the relationship between *D*_EANM_ and mean *D*_image_ without PVC (${\overline{D}}_{\mathrm{image}}$). There was a strong correlation (Pearson’s correlation coefficient = 0.82) between the conventional method (*D*_EANM_) and image-based method (${\overline{D}}_{\mathrm{image}}$), using the regression equation: ${\overline{D}}_{\mathrm{image}}=0.47\times {D}_{\mathrm{EANM}}+4.89$ (95% confidence intervals (CI) of slope and *y*-intercept = 0.37–2.19 (*p* < 0.001) and 0.56–47.59 (*p* = 0.03), respectively). Figure 3b shows the Bland-Altman plot between *D*_EANM_ and ${\overline{D}}_{\mathrm{image}}$. There were fixed bias (paired *t* test, *p* < 0.001) and proportional bias (Pearson’s correlation coefficient = − 0.72 [95% CI = − 0.84 to − 0.55]). Fixed bias was − 97.7 Gy (95% CI = − 107.9 to − 87.4), that is, ${\overline{D}}_{\mathrm{image}}$ was lower than *D*_EANM_ in the same patient.

**Fig. 4:**
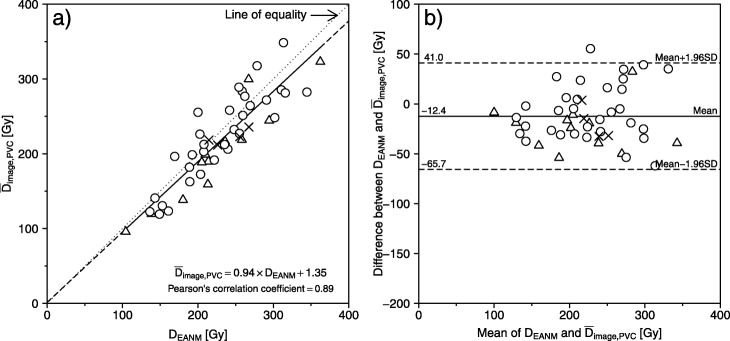
**a** Relationship between EANM-based absorbed dose (*D*_EANM_) and image-based absorbed dose with PVC (${\overline{D}}_{\mathrm{image},\mathrm{PVC}}$), using the regression equation: ${\overline{D}}_{\mathrm{image},\mathrm{PVC}}=0.94\times {D}_{\mathrm{EANM}}+1.35$ (95% CIs of slope and *y*-intercept = 0.80–1.08 and − 32.2–34.8, respectively]. **b** The Bland-Altman analysis was performed between *D*_EANM_ and ${\overline{D}}_{\mathrm{image},\mathrm{PVC}}$. There was also fixed bias (− 12.4 Gy [95% CI = − 20.3 to − 4.4]) but no proportional bias (Pearson’s correlation coefficient = 0.11 [95% CI = − 0.17–0.39]). Note that each symbol in the figure represents three therapeutic outcomes: open circle, hypothyroidism; open triangle, euthyroidism; and cross, hyperthyroidism

Similarly, Fig. 4a shows the relationship of *D*_EANM_ and mean *D*_image_ with PVC (${\overline{D}}_{\mathrm{image},\mathrm{PVC}}$). There was also a strong correlation (Pearson’s correlation coefficient = 0.89) between both methods with the regression equation: ${\overline{D}}_{\mathrm{image},\mathrm{PVC}}=0.94\times {D}_{\mathrm{EANM}}+1.35$ (95% confidence intervals (CI) of slope and *y*-intercept = 0.80–1.08 (*p* < 0.001) and − 32.2–34.8 (*p* = 0.94), respectively). Figure 4b shows the Bland-Altman plot between *D*_EANM_ and ${\overline{D}}_{\mathrm{image},\mathrm{PVC}}$ in three therapeutic outcomes. There was also fixed bias (paired *t* test, *p* < 0.01) but no proportional bias (Pearson’s correlation coefficient = 0.11 (95% CI = − 0.17–0.39]). The fixed bias was − 12.4 Gy (95% CI = − 20.3 to − 4.4); ${\overline{D}}_{\mathrm{image},\mathrm{PVC}}$ was almost equivalent to *D*_EANM_ in the same patient. Twelve of 16 cases whose therapeutic outcomes were euthyroidism or hyperthyroidism fell below the regression line in Fig. 4.

**Fig. 5:**
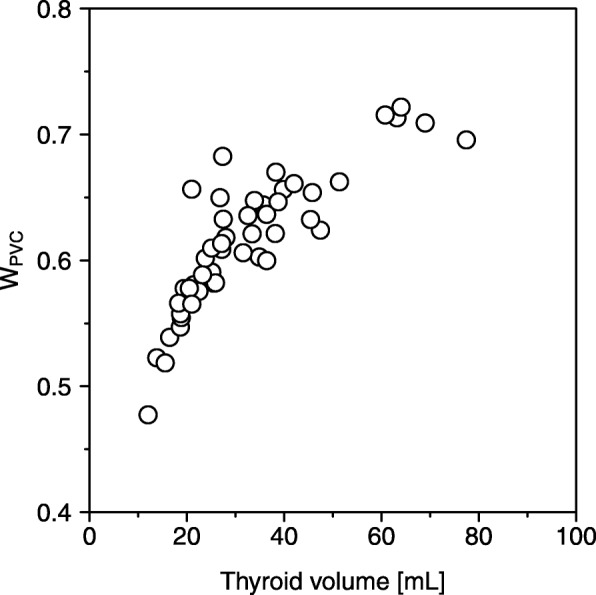
Relationship between weighting factor, *W*_PVC_, used for partial volume correction and thyroid volume

In the scan and reconstruction conditions of this study, the spatial resolution (full width at half maximum [FWHM]) in the *x*, *y*, and *z* directions were 11.0, 10.4, and 11.0 mm, respectively. The weighting factor, *W*_PVC_, for each case can be calculated from the 3D point spread function and the thyroid volume in the SPECT image (Eq. 8). The relationship between the *W*_PVC_ calculated using the GTM method (range = 0.45–0.80) and thyroid volume for each case is shown in Fig. 5.

Table 2 shows the relationship between therapeutic outcomes and thyroid-absorbed dose calculated from each method (*D*_EANM_, ${\overline{D}}_{\mathrm{image}}$, and ${\overline{D}}_{\mathrm{image},\mathrm{PVC}}$). Thirty-two cases (67%) analyzed in this study eventually had hypothyroidism. However, there was no significant difference between the three therapeutic outcomes with any of the methods.

**Table 2:** Relationship between therapeutic outcomes and thyroid-absorbed dose calculated from each method

	Hypothyroidism	Euthyroidism	Hyperthyroidism
Cases	32 (67%)	12 (25%)	4 (8%)
*D*_EANM_ [Gy]^a^	231.4 ± 55.9 (136.3–344.6)	223.6 ± 68.5 (104.3–362.3)	241.0 ± 24.7 (214.6–267.5)
${\overline{D}}_{\mathrm{image}}$ [Gy]^b^	137.5 ± 29.7 (85.4–200.7)	117.2 ± 39.5 (53.1–186.2)	139.3 ± 12.4 (126.0–155.9)
${\overline{D}}_{\mathrm{image},\mathrm{PVC}}$ [Gy]^c^	224.3 ± 60.0 (119.3–348.6)	199.4 ± 67.9 (95.7–323.0)	222.1 ± 10.2 (211.6–235.8)

*Data are expressed as mean ± 1 standard deviation (SD), if appropriate, as maximum and minimum values

^a^Hypo- vs Eu-, *p* = 0.785; Eu- vs Hyper-, *p* = 0.379; Hypo- vs Hyper-, *p* = 0.645

^b^Hypo- vs Eu-, *p* = 0.063; Eu- vs Hyper-, *p* = 0.133; Hypo- vs Hyper-, *p* = 0.981

^c^Hypo- vs Eu-, *p* = 0.234; Eu- vs Hyper-, *p* = 0.317; Hypo- vs Hyper-, *p* = 0.903

(Bonferroni-corrected Wilcoxon-Mann-Whitney test)

## Discussion

Various thyroid-absorbed dose calculation methods have been devised previously [6–8, 17]; however, none was image-based, and all assumed that iodine uptake by the thyroid is homogeneous. Furthermore, these calculation methods were designed to determine administered radioactivity before radioiodine therapy. Therefore, there is no effective method for determining absorbed doses after radioiodine therapy. The pre-therapeutic dosimetry described by Marinelli et al. or in the EANM guidelines can only be estimated [8–10]. Iodine uptake by the thyroid does not always conform to the models used to determine their calculation formulae. As a result, there is a difference between the *estimated* and *actual* absorbed doses. In contrast to these methods, we herein examined the effectiveness of the post-therapeutic dosimetry based on quantitative SPECT imaging and thyroid biokinetics by expanding the MIRD’s guidelines for voxel dosimetry. Because this method uses RIU values and SPECT images taken at the time of treatment, the distribution of iodine-131 in the thyroid is directly reflected in the absorbed dose distribution, and it is possible to visualize local iodine uptake in the thyroid. Although previous studies have also utilized SPECT imaging to calculate absorbed doses for oncology [11, 12], no reports demonstrate its application to Graves’ disease.

From post-therapeutic SPECT images, we observed that iodine uptake and absorbed dose may not always be homogeneously distributed in the thyroid (Fig. 1). Generally, Graves’ disease increases iodine uptake of the thyroid in a diffuse manner [1]. Therefore, a homogenous distribution of radioactivity is expected, and as such is a presumption when calculating absorbed dose using the conventional method. However, we noted several cases in which the absorbed dose was heterogeneous (Fig. 1), which supports the possibility that uptake of iodine differs locally in Graves’ disease. These local differences in iodine uptake would therefore widen the range of absorbed doses. Post-therapeutic image-based dosimetry could help to reduce inaccuracies in thyroid dose calculations by accounting for local differences in iodine uptake.

Furthermore, the absorbed dose was widely distributed for each patient (Fig. 2). One reason for this observation could be explained by the partial volume effect caused by the low spatial resolution of SPECT; in this study, several methods were used to suppress the degradation of spatial resolution, including OSEM-3D, increasing the update number (subset number × iteration number), and the absence of a post-filter. However, it was not possible to completely eliminate the influence of spatial resolution deterioration. Therefore, the broad distribution of absorbed doses was likely influenced by the partial volume effect to some extent. Moreover, the mean *D*_image_ decreased 0.45–0.80 times without PVC depending on the thyroid volume (Fig. 5). With the deterioration of spatial resolution, SPECT counts in the thyroid spill-out of this region. As a result, the dose distribution is skewed towards lower values due to the partial volume effect. Analyzing the results with and without PVC, PVC is necessary to compare *D*_EANM_ as pre-therapeutic dosimetry and *D*_image_ as post-therapeutic dosimetry. Prior to this study, we also tried voxel-by-voxel PVC by the Lucy-Richardson method [24], but this method resulted in Gibbs artifacts on the SPECT image. We determined that the image-based absorbed dose should not be calculated from images with Gibbs artifacts, and we have abandoned the application of this method. Using higher-resolution SPECT images using image restoration or super-resolution techniques could, therefore, result in more accurate determinations of absorbed dose distribution.

Absorbed doses determined using the conventional EANM-based and image-based methods (*D*_EANM_ and ${\overline{D}}_{\mathrm{image},\mathrm{PVC}}$, respectively) had a strong correlation, but there was fixed bias (Fig. 4). This correlation implies that post-therapeutic image-based dosimetry provides an appropriate alternative to standard pre-therapeutic dosimetry to evaluate dose response. This also accounts for heterogeneity in iodine uptake by the thyroid by providing additional information, including dose distribution.

Both the rate of therapeutic outcomes and the likelihood of developing hypothyroidism increase with increasing thyroid-absorbed radioactivity doses [4, 5, 20, 25]. However, cutoff values for absorbed dose have yet to be established for radioiodine therapy for Graves’ disease, possibly because there has previously been no clear way to determine absorbed doses after treatment. Using the post-therapeutic image-based method here (i.e., absorbed dose distribution and ${\overline{D}}_{\mathrm{image},\mathrm{PVC}}$), it may be possible to identify a clear relationship between therapeutic outcomes and dose distribution in the thyroid. Our hospital aims to treat Graves’ disease with radioiodine therapy, achieving iatrogenic hypothyroidism that has been completely compensated to the euthyroid state with oral levothyroxine. Therefore, the administered radioactivity or prescribed absorbed dose to patients with Graves’ disease has been set higher. Many cases whose therapeutic outcomes were euthyroidism or hyperthyroidism fell below the regression line (Figs. 3 and 4). This might be due to the difference between *estimated* and *actual* absorbed doses. However, there was no significant difference between the three therapeutic outcomes with any of the methods (Table 2). The limitation of this study is that we could not identify the relationship between image-based absorbed doses and therapeutic outcomes because of the retrospective nature of the study. Additional prospective studies are required to further investigate the relationship between post-therapeutic image-based dosimetry and therapeutic outcomes.

Another limitation is the necessity for pre-therapeutic measurements to obtain the individual biokinetic properties in patients as only a single SPECT scan is acquired. According to previous studies [20, 21], we assumed little difference between RIU at therapeutic and test doses. Conversely, there were also reports that the biokinetics of iodine-131 at treatment differed from that at uptake test [26, 27]. Canzi et al. reported that iodine-131 uptake at treatment was reduced by 12% due to the stunning effect of iodine-131 therapeutic activity [27]. Assuming the same biokinetic behavior between pre- and post-therapy, each voxel may increase in uncertainty or accrue a systematic error in the absorbed doses obtained. To resolve this uncertainty, we need to carry out multiple SPECT examinations during radioiodine therapy to directly reflect the biokinetics at treatment.

In our study, the administered radioactivity in Graves’ disease was 500 MBq or less. Therefore, we considered that count loss minimally affected this situation, and we did not need to perform the dead time correction. However, in the most recent paper by Gregory et al. [28], they measured the resolving time τ with the same type of SPECT/CT system used in this study (3.796 × 10^−6^ s). Taking into account that the mean count rate of projection right above the thyroid in each patient was 10.7 ± 3.6 kcps in the 15% main window centered at 364 keV, the dead time correction factor for the non-paralyzable model, *D*_τ_, calculated from Eq. 10 is 1.04 ± 0.01 (range = 1.01–1.07):

10 $$ {D}_{\tau }=\frac{R_{\mathrm{T}}}{R_{\mathrm{M}}}=\frac{1}{\left(1-{R}_{\mathrm{M}}\tau \right)} $$

where *R*_T_ is the true count rates and *R*_M_ is the measured count rates. Not performing the dead time correction is a limitation of this study.

Assuming the dead time correction is simply performed by multiplying *D*_τ_ by ${\overline{D}}_{\mathrm{image},\mathrm{PVC}}$ for each patient, the regression equation between the dead time corrected ${\overline{D}}_{\mathrm{image},\mathrm{PVC}}$, namely ${\overline{D}}_{\mathrm{image},\mathrm{PVC}}^{\prime }$ and *D*_EANM_ is ${\overline{D}}_{\mathrm{image},\mathrm{PVC}}^{\prime }$ = 0.98 × *D*_EANM_ + 1.23. With this correction, the fixed bias between *D*_EANM_ and ${\overline{D}}_{\mathrm{image},\mathrm{PVC}}^{\prime }$ was also eliminated (paired *t* test, *p* = 0.454). The fixed bias between *D*_EANM_ and ${\overline{D}}_{\mathrm{image},\mathrm{PVC}}$ could be eliminated by performing the strict dead time correction.

We must also consider the option of performing image-based dosimetry before treatment, although we must be aware that performing this dosimetry may conflict with dietary iodine restrictions. Our country is classified as an iodine-sufficient area, and all patients are subjected to strict dietary iodine restriction before treatment. Thus, pre-therapeutic image-based dosimetry is not practical and post-therapeutic image-based dosimetry proves more reasonable.

## Conclusion

In this study, we validated the feasibility of the post-therapeutic image-based dosimetry for Graves’ disease, applying quantitative SPECT, voxel S values, and RIU values. Post-therapeutic image-based thyroid-absorbed doses were consistent with those of pre-therapeutic EANM method-based absorbed doses. In addition, a post-therapeutic image-based method had the advantage of being able to visualize thyroid iodine distribution and hence determine local absorbed dose distributions at the time of treatment. From these points, we suggest that post-therapeutic image-based dosimetry could provide an appropriate alternative to standard pre-therapeutic dosimetry for dose-response evaluation. Future studies are required to improve the accuracy of dosimetry and identify the relationship between thyroid-absorbed dose at the time of radioiodine therapy and therapeutic outcomes.

## Data Availability

The datasets used and/or analyzed during the current study are available from the corresponding author on reasonable request.

## Abbreviations

CI: Confidence interval
EANM: European Association of Nuclear Medicine
FWHM: Full width at half maximum
GTM: Geometry transfer matrix
HE: High energy
kcps: Kilocounts per second
MIRD: Medical Internal Radiation Dose Committee
OSEM-3D: Ordered subset expectation maximization with depth-dependent three-dimensional resolution recovery
PVC: Partial volume correction
RIU: Radioiodine uptake
RSF: Regional spread function
SPECT: Single-photon emission computed tomography
VOI: Volume of interest
